# A multi-factor model for caspase degradome prediction

**DOI:** 10.1186/1471-2164-10-S3-S6

**Published:** 2009-12-03

**Authors:** Lawrence JK Wee, Joo Chuan Tong, Tin Wee Tan, Shoba Ranganathan

**Affiliations:** 1 Department of Biochemistry, Yong Loo Lin School of Medicine, National University of Singapore, Singapore; 2 Singapore Immunology Network, Singapore; 3 Institute for Infocomm Research, 1 Fusionpolis Way, #21-01 Connexis, Singapore 138632; 4 Department of Chemistry and Biomolecular Sciences & ARC Centre of Excellence in Bioinformatics, Macquarie University, Sydney, Australia

## Abstract

**Background:**

Caspases belong to a class of cysteine proteases which function as critical effectors in cellular processes such as apoptosis and inflammation by cleaving substrates immediately after unique tetrapeptide sites. With hundreds of reported substrates and many more expected to be discovered, the elucidation of the caspase degradome will be an important milestone in the study of these proteases in human health and disease. Several computational methods for predicting caspase cleavage sites have been developed recently for identifying potential substrates. However, as most of these methods are based primarily on the detection of the tetrapeptide cleavage sites - a factor necessary but not sufficient for predicting *in vivo *substrate cleavage - prediction outcomes will inevitably include many false positives.

**Results:**

In this paper, we show that structural factors such as the presence of disorder and solvent exposure in the vicinity of the cleavage site are important and can be used to enhance results from cleavage site prediction. We constructed a two-step model incorporating cleavage site prediction and these factors to predict caspase substrates. Sequences are first predicted for cleavage sites using CASVM or GraBCas. Predicted cleavage sites are then scored, ranked and filtered against a cut-off based on their propensities for locating in disordered and solvent exposed regions. Using an independent dataset of caspase substrates, the model was shown to achieve greater positive predictive values compared to CASVM or GraBCas alone, and was able to reduce the false positives pool by up to 13% and 53% respectively while retaining all true positives. We applied our prediction model on the family of receptor tyrosine kinases (RTKs) and highlighted several members as potential caspase targets. The results suggest that RTKs may be generally regulated by caspase cleavage and in some cases, promote the induction of apoptotic cell death - a function distinct from their role as transducers of survival and growth signals.

**Conclusion:**

As a step towards the prediction of *in vivo *caspase substrates, we have developed an accurate method incorporating cleavage site prediction and structural factors. The multi-factor model augments existing methods and complements experimental efforts to define the caspase degradome on the systems-wide basis.

## Background

It is increasingly being recognized that proteolytic processing, or the specific and limited cleavage of proteins by enzymes called proteases, represents an important mechanism for cellular control in all living organisms [1]. Elucidating the protease degradome - the complete substrate repertoire of the protease in a cell, tissue or organism - at the systems level will unravel important clues on protease function across biological pathways and inter-connections with other protease systems. However, the experimental discovery and validation of bona fide protease substrates require time consuming and laborious efforts. As such, computational tools for the prediction of protease degradomes will complement these efforts.

In recent years, much work had been done on the prediction of the substrates of caspases - a unique class of cysteine proteases which function as critical effectors of apoptosis, inflammation and other important cellular processes [2-4]. Caspases recognizes highly specific tetrapeptide motifs (denoted as P_4_-P_3_-P_2_-P_1_) and cleave substrates after the requisite Asp residue at P_1 _[5]. Substrates of caspases belong to a myriad of protein classes such as structural elements of the cytoplasm and the nucleus, components of the DNA repair machinery, protein kinases, GTPases and viral structural proteins [6,7]. Hundreds of caspase substrates have been reported and many more are expected to be discovered. Most of the current approaches for caspase substrates prediction are primarily based on the detection of cleavage sites on proteins using information encoded within the tetrapeptide motifs (reviewed in [8]). While the identification of the specific cleavage site on the primary sequence of a protein is necessary for substrate prediction, it is intuitive that the final proteolytic cleavage of a protein *in vivo *is contingent on a multitude of other factors in addition to the presence of cleavage sites. Based on our analysis on a dataset of 176 experimentally verified caspase substrates (details are available in Additional File 1), we found that 80% of substrates contain at least one other identical caspase cleavage site sequence which is not reported as a true cleavage site in the literature. Identical cleavage site sequences in Tpr (DDED^2117^) [9], p28BAP31 (AAVD^163^) [10-12], golgin 160 (SEVD^311^) [13], Topo I (PEDD^123^) [14,15] and heterogeneous nuclear ribonucleoparticle C1/C2 (GEDD^305^) [16], are located at two distinct positions on the respective protein but only one was reported to be cleaved. Indeed, it is suggested that conformation of the local structure of the cleavage site and not just the primary sequence alone is required for protease cleavage. Unstructured regions of substrates appear to be more susceptible to cleavage than regions of secondary structure (helices and *β*-sheets) [8]. Also, the structures of a number of *in vivo *caspase substrates such as Bid [17,18], ICAD [19,20] and pro-caspase-7 [21,22] suggest that caspase cleavage sites have a preference for location within disordered or unstructured extended loops, in line with observations on protease substrates in general [23]. It is also suggested that the location of cleavage sites is critical for substrate cleavage - a potential cleavage site needs to be located at the surface of the substrate, rather than within the hydrophobic core of the protein, in order to be accessible to the protease active site [24].

In this context, we are motivated to explore the integration of these structural factors with cleavage site prediction to better predict caspase substrates. We report that caspase cleavage sites have a higher propensity to locate in unstructured and solvent exposed regions on the substrate compared to non-cleavage sites. We propose a two-step, multi-factor model incorporating these factors together with the caspase cleavage sites prediction tools to augment the prediction of caspase substrates. When CASVM [25,26] and GraBCas [27] were integrated into the model, prediction results were shown to achieve greater positive predictive values compared to CASVM or GraBCas alone. The model was able to reduce the false positive pool by up to 13% and 53% respectively while retaining all true positives. In addition, we applied our prediction model on the family of receptor tyrosine kinases (RTKs) and highlighted several members as potential caspase targets. The results suggest that RTKs may be generally regulated by caspase cleavage, and in some cases, promote the induction of apoptotic cell death - a function distinct from their role as transducers of survival and growth signals.

## Results

### Structural tendencies of caspase cleavage sites

**Figure 1:**
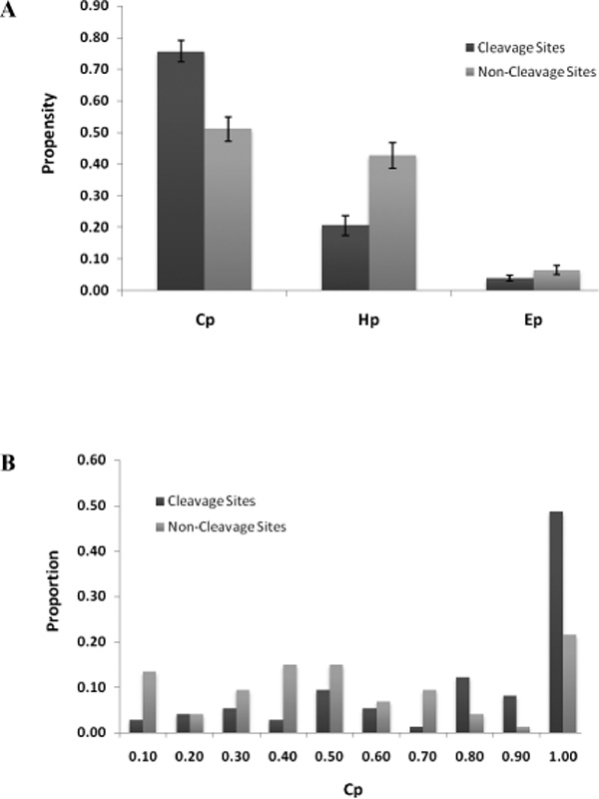
**Propensity for cleavage sites to occur in secondary structures**. A. Propensities for different secondary structure elements (*coils*, C_p_; *helices*, H_p_; *β-strands*, E_p_) were measured for 24-mer subsequences with or without caspase cleavage sites (labelled "*cleavage sites*" and "*non-cleavage sites*" respectively). B. Distribution of "*cleavage sites*" and "*non-cleavage sites*" subsequences to C_p _bins. Each C_p _bin (0.10, 0.20...1.00) was allocated a proportion of sequences with C_p _scores falling within the bin range (0-0.10, 0.11-0.19...0.91-1.00).

We explored the likelihood for caspase cleavage sites to locate in unstructured segments spanning across the tetrapeptide recognition sequence on the folded protein. To measure the propensities for various secondary structures across the cleavage site region, an analysis dataset of 24-mer peptide subsequences comprising of the tetrapeptide cleavage sites or randomly selected tetrapeptide sequences on the same protein plus upstream ten residues up to P_14 _and downstream ten residues up to P_10_' was constructed. Secondary structural elements in these subsequences were predicted using SABLE II protein structure prediction server (see Materials and methods for details). The propensities for secondary structures were quantified as H_p_, E_p _and C_p _scores for helices, *β*-strands and coils respectively. As shown in Figure 1A, the propensity for coils was significantly greater for cleavage site subsequences compared to non-cleavage site subsequences (mean C_p _= 0.76 versus C_p _= 0.51 respectively, P-value < 0.01), while the propensity for helices was greater for non-cleavage site subsequences compared to cleavage site subsequences (mean H_p _= 0.43 versus H_p _= 0.21 respectively, P-value < 0.01). The distribution of cleavage site subsequences and non-cleavage site subsequences were further analyzed across C_p _bins (Figure 1B). It was shown that cleavage site subsequences were distributed more frequently to bins of higher C_p _scores compared to non-cleavage site subsequences. Most cleavage site subsequences were confined to bins 0.8-1.0 while a greater proportion of non-cleavage site subsequences were distributed to bins less than 0.8.

**Figure 2:**
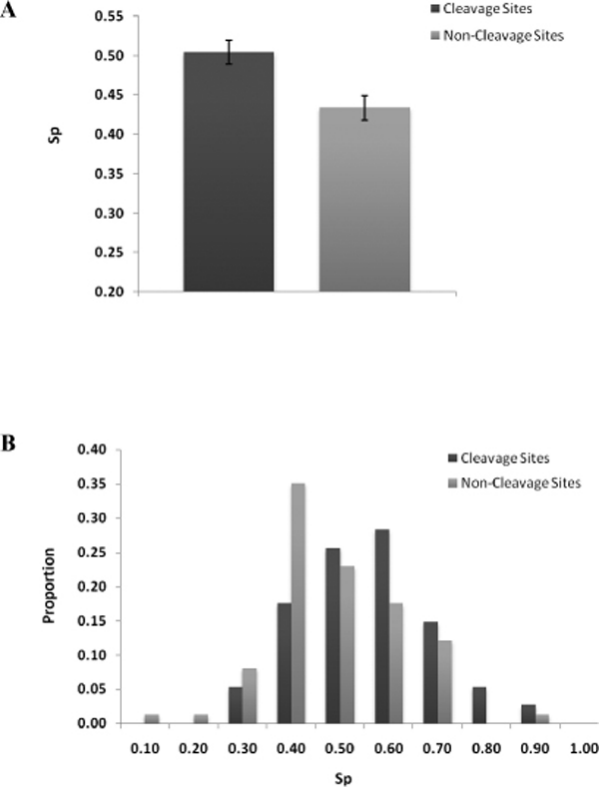
**Propensity for cleavage sites to occur in solvent accessible locations**. A. Propensities for solvent accessibilities (Sp) were measured for 24-mer subsequences with or without caspase cleavage sites (labelled "cleavage sites" and "non-cleavage sites" respectively). B. Distribution of "cleavage sites" and "non-cleavage sites" subsequences to Sp bins. Each Sp bin (0.10, 0.20...1.00) was allocated a proportion of sequences with Sp scores falling within the bin range (0-0.10, 0.11-0.19...0.91-1.00).

**Figure 3:**
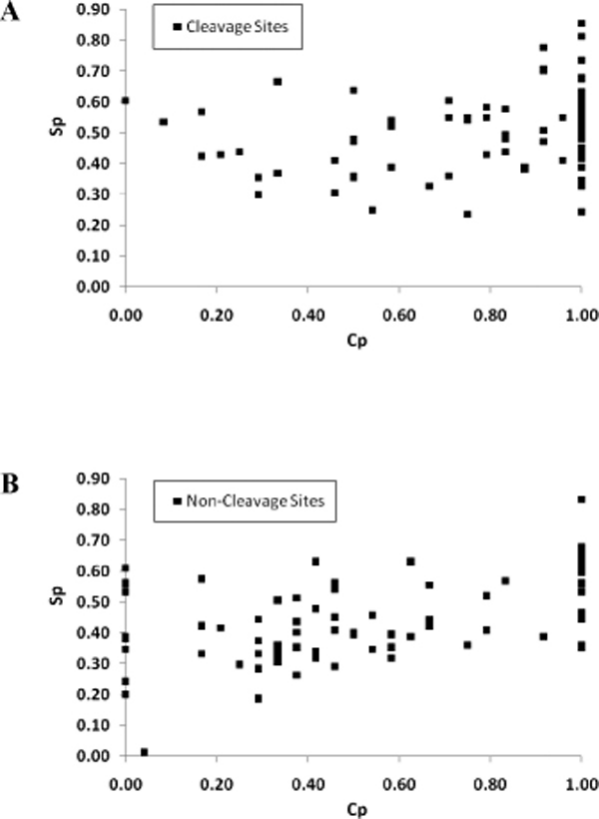
**Scatter plots of Sp and Cp values. **Each data point corresponds to the Sp and Cp values of a single 24-mer subsequence of A. cleavage sites and B. non-cleavage sites.

Next, we asked if cleavage sites would preferentially locate in solvent exposed regions of substrates. The solvent accessibilities of the 24-mer peptide subsequences from the analysis dataset were predicted using the SABLE II server. S_p_, a composite variable for measuring the propensity for solvent exposure was calculated for all subsequences. As shown in Figure 2A, cleavage site subsequences were more exposed to solvent compared to non-cleavage site subsequences (mean S_p _= 0.50 versus S_p _= 0.43 respectively, P-value < 0.01). When the distribution of the subsequences were analyzed across S_p _bins (Figure 2B), both cleavage and non-cleavage site subsequences were found to be increasingly distributed into regions of greater solvent exposure as S_p _increases from 0 to 0.40. However, distribution of non-cleavage site subsequences peaked at S_p _of 0.40, while the distribution for cleavage site subsequences peaked at 0.60, before falling off along the higher S_p _bins values. Taken together, these results suggest that caspase cleavage sites tend to locate in unstructured and solvent exposed regions on substrates. Interestingly, we further noted a positive correlation (overall correlation coefficient, *r *= 0.43) between S_p _and C_p _values across all cleavage site and non-cleavage site subsequences (Figure 3A and 3B), further confirming the tendency for cleavage sites to locate in regions with both characteristics and vice versa.

### Multi-factor model for prediction of caspase substrates

**Figure 4:**
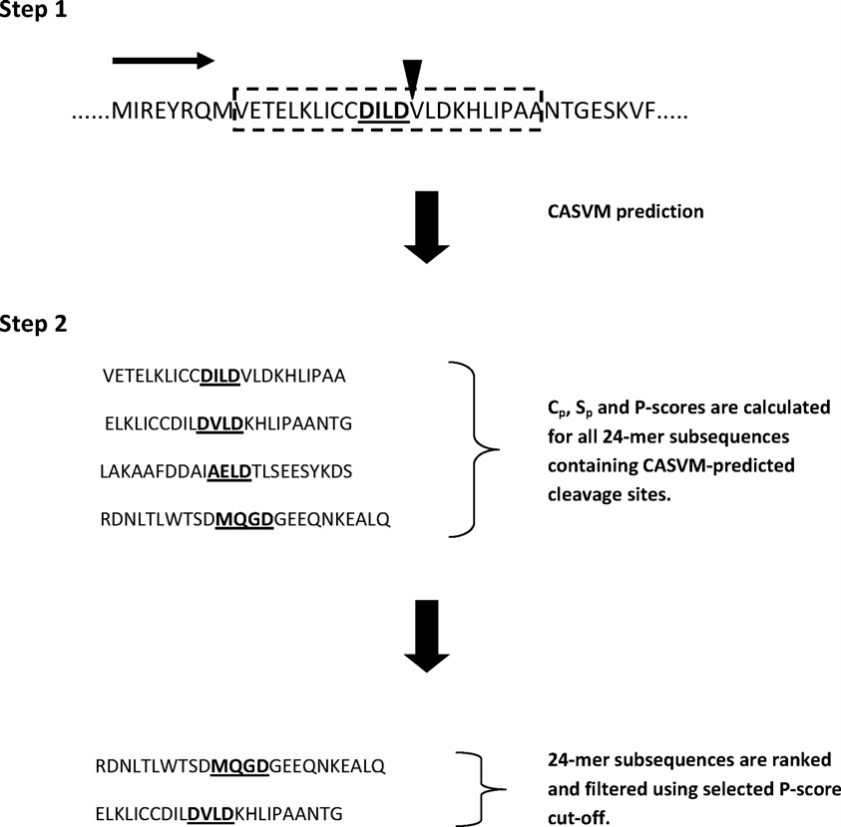
**Schematic diagram of the multi-factor model for caspase substrate prediction**. Step1: A window scans the entire protein (example used: 14-3-3, Uniprot ID: P31946) for potential caspase cleavage sites (bold, underlined, P1 residue to the left of inverted triangle) using a caspase cleavage site prediction tool (example used: a 24 residue scanning window from CASVM). Step 2: 24-mer subsequences containing predicted cleavage sites with flanking ten residues downstream and upstream from the tetrapeptide sequence (P14 to P10') are constructed. Cp, Sp and P-score are calculated for all subsequences. Cleavage sites in subsequences with P-score above the assigned cut-off are selected.

As noted earlier, the distinguishing factor for caspase substrate cleavage is the presence of specific tetrapeptide sequences on the substrate protein. However, the usage of tetrapeptide specificities solely for detection of *in vivo *substrate cleavage is likely to produce a high percentage of false positives. With the exception of GraBCas and CASVM, computational tools for caspase cleavage sites prediction rely strictly on these tetrapeptide sequences in their algorithms. The GraBCas software utilizes position specific scoring matrices (PSSM) and accounts for the tetrapeptide sequences and the downstream P_1_' and P_2_' residues. CASVM was developed recently by our group, based on the support vector machine (SVM) algorithm where sequence windows of varying lengths flanking the cleavage sites are employed. Given that bona fide cleavage sites tend to locate in unstructured and solvent exposed regions, we hypothesized if structural factors can be used to improve the prediction of caspase substrates by filtering out predicted cleavage site sequences with unfavourable structural characteristics. Accordingly, we propose a prediction model for caspase substrates using a two-step approach to encapsulate these factors (Figure 4). In the first step, the entire protein is scanned for potential caspase cleavage sites using a caspase cleavage site prediction tool (a 24-mer scanning window in CASVM is used as example). In the second step, 24-mer subsequences - comprising of the predicted cleavage sites with flanking ten residues downstream and upstream from the tetrapeptide sequence (P_14 _to P_10_') - were constructed and C_p_ and S_p _values calculated. The C_p _and S_p _values are combined into the variable P-score, which quantifies the net propensities for both unstructured regions and solvent exposure in the subsequences. Using an assigned P-score cut-off, cleavage sites in subsequences scoring above the cut-off are selected while the rest are eliminated.

**Figure 5:**
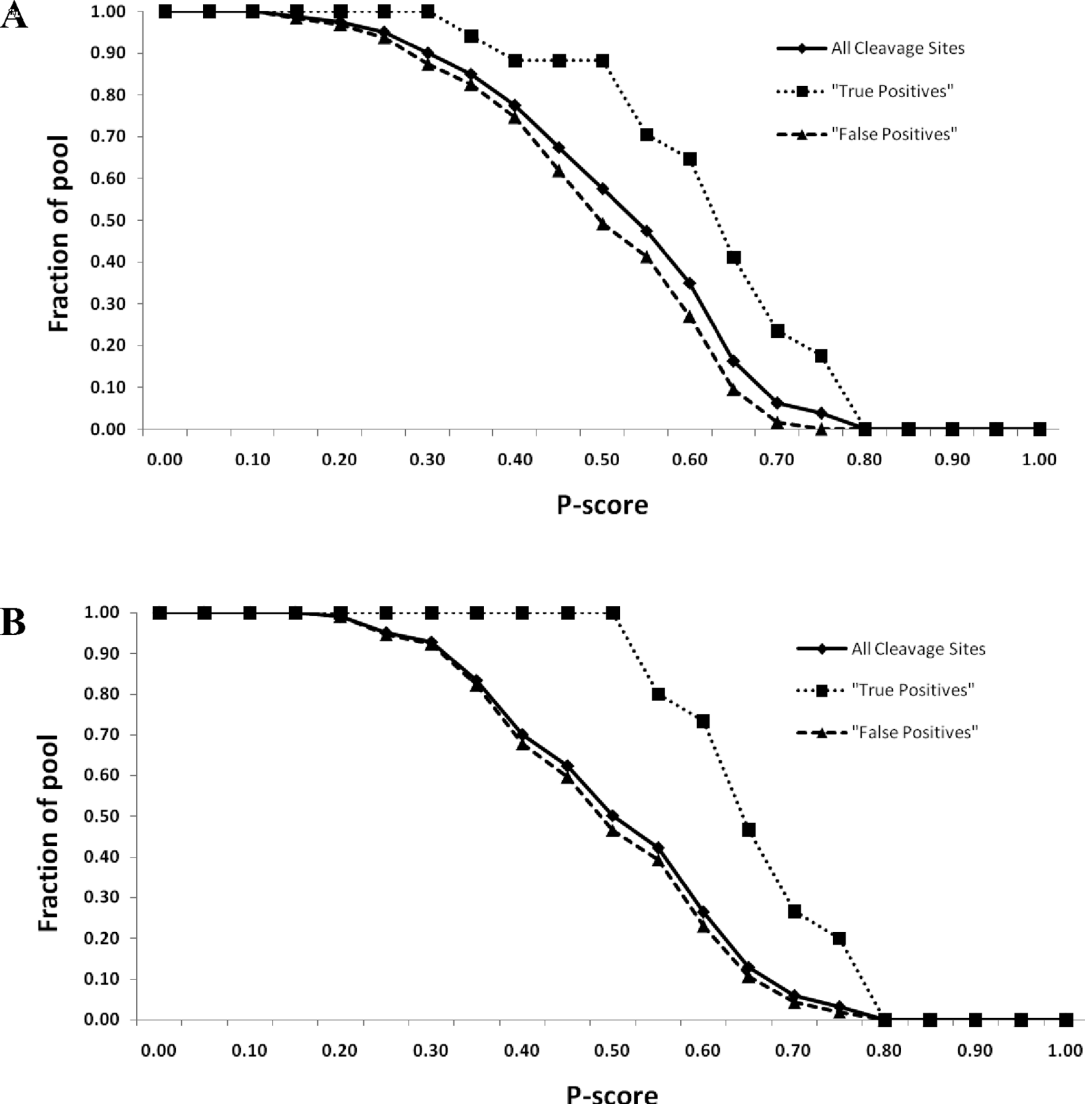
**Result of model prediction on test dataset**. A. CASVM predicted cleavage sites were assigned to pools containing "true positives" or "false positives". Fractions of cleavage sites in their respective pools (vertical axis) with P-scores above the cut-offs (horizontal axis) were measured. B. GraBCas predicted cleavage sites were assigned to pools containing "true positives" or "false positives". Fractions of cleavage sites in their respective pools (vertical axis) with P-scores above the cut-offs (horizontal axis) were measured.

To measure effectiveness of the model in improving caspase substrate prediction, CASVM and GraBCas were implemented into the model and tested with an independent dataset. In our earlier work, we reported that CASVM, using the support vector machines algorithm, achieved an accuracy of 89% when tested on an independent dataset of caspase cleavage sites. GraBCas, on the other hand, utilizes position-specific scoring matrices for scoring and selecting potential tetrapeptide sequences and achieved an accuracy of 70% (at cut-off of 0.1) using the same dataset. Stepping through the first step of the model, CASVM predicted 80 tetrapeptide sequences across all 14 substrates as potential caspase cleavage sites, inclusive of all 17 cleavage site sequences from the test dataset, while GraBCas predicted 223 tetrapeptide sequences which included 15 of the 17 cleavage sites. At the second step, subsequences were constructed as described and calculated for their P-score values. Retention of subsequences at P-score cut-off values from 0 to 1.00 was measured. Subsequences containing cleavage sites were assigned as "true positives" and all other subsequences were assigned as "false positives". As shown in Figure 5A, all CASVM predicted subsequences were retained at P-score of 0 but were steadily eliminated as the cut-off progressed to 1.00. In addition, from P-score cut-off of 0.15 to 0.80, proportionately more true positives were retained compared to the false positives. At P-score of 0.50, about 88% of true positives were retained but more than half of the false positives were eliminated. Similarly, all GraBCas predicted subsequences were eliminated in tandem with increasing P-score cut-offs and proportionately more true positives were retained from P-score cut-off of 0.25 to 0.80 compared to the false positives (Figure 5B). At P-score of 0.50, 100% of true positives were retained but more than half of the false positives were eliminated. These metrics suggest that a defined range of P-score cut-offs can be used to discriminate and filter the false positives from cleavage site prediction. Interestingly, the GraBCas results showed that false positives were filtered off at P-score cut-off of 0.20 onwards while true positives were eliminated only at a higher cut-off of 0.55. When CASVM was used, false positives were filtered off from P-score cut-off of 0.15 and true positives from 0.35. The higher P-score cut-off for true positives compared to the false positives in both cases further suggest that a notable proportion of false positives - up to 13% and 53% in CASVM and GraBCas models respectively - can be eliminated without a reduction in the size of the original pool of true positives. In addition, from the prediction results, we measured the positive predictive value (PPV) at each P-score cut-off for models implementing CASVM and GraBCas (Table 1, denoted under *CASVM-model *and *GraBCas-model *respectively). The usage of CASVM alone obtained a PPV of 21.25%, while GraBCas achieved 6.73% (results are given under P-score cut-off of 0). Notably, while the usage of either methods on its own could predict most, if not all of the cleavage sites, the relatively low PPV values intuitively suggest a proportionately greater number of false positive cleavage sites. However, the proportion of false positives can be reduced in the multi-factor model implementing CASVM or GraBCas since higher P-score cut-offs were correlated with higher PPV values for both cases. Taken together, these results indicate that the combination of structural factors such as unstructured regions and solvent exposure can be used to refine results from cleavage site prediction by specific filtering of false positives cleavage sites at defined P-score cut-off levels. The reduction of the false positives cleavage sites will lead to more accurate prediction of caspase substrates.

**Table 1:** Positive predictive values (PPV) of model prediction at various P-score cut-offs.

	**PPV (%)**^1^	
	
P-score	*CASVM-Model*	*GraBCas-Model*
0.00	21.25	6.73
0.05	21.25	6.73
0.10	21.25	6.73
0.15	21.52	6.73
0.20	21.79	6.79
0.25	22.37	7.08
0.30	23.61	7.25
0.35	23.53	8.06
0.40	24.19	9.62
0.45	27.78	10.79
0.50	32.61	13.39
0.55	31.58	12.77
0.60	39.29	18.64
0.65	53.85	24.14
0.70	80.00	30.77
0.75	100.00	42.86

^1^PPV calculations are limited to P-score cut-off of 0.75 as all predicted cleavage sites were eliminated beyond this point for both models. *CASVM-Model *and *GraBCas-Model *indicate the use of either CASVM or GraBCas at the first step of the model.

## Prediction of caspase cleavage of RTKs

Receptor tyrosine kinases (RTKs) belong to a sub-class of the protein kinase superfamily which function as plasma membrane-bound receptors transducing extracellular signals mediating cell survival, proliferation, embryonic development, adult homeostasis and many other critical processes [28]. As RTK activity in resting, normal cells is tightly controlled, mutations or structural aberrations in RTKs were shown to convert them to potent oncoproteins, contributing to the development and progression of many cancers. Interestingly, recent studies have implicated several members of the receptor tyrosine kinase (RTK) family - as such EGFR [29,30], Erbb2 [31-33], MET [34-36], RET [37] and ALK [38] - as proteolytic targets of caspases during apoptosis. Given the pervasive role of RTKs in cell survival and proliferation pathways and their implications in diseases such as cancer, it is tempting to speculate if RTKs may be generally regulated by caspase activity and if many other RTKs remain hitherto undiscovered downstream targets of caspases. Accordingly, we applied the multi-factor model to predict for potential caspase substrates among the members of the RTK family and analyzed the results.

The complete repertoire of RTK sequences - 52 members across 16 sub-families, as listed in the KEGG database [39] - was retrieved from Uniprot database [40] and predicted for potential caspase substrates using the multi-factor model. Protein sequences of RTKs were submitted to the CASVM server under default settings and predicted cleavage sites were scored for their propensities for solvent exposure and unstructured regions as described earlier. Predicted cleavage sites with equal or less than the P-score cut-off of 0.3 were highlighted (results are listed in Additional File 2; P-score of 0.3 was chosen as it represented the highest possible cut-off before true positives were filtered as shown in Figure 5A). The results showed that all RTKs were predicted to possess caspase cleavage sites which are distributed throughout the length of the extracellular and intracellular regions of the RTKs. About 92% of all RTKs (48/52) possess cleavage sites on the intracellular region while about 98% (51/52) contain extracellular cleavage sites. While predicted cleavage sites localize throughout the length of the receptors, notable trends in the distribution of predicted sites imply functional significance downstream of caspase cleavage. A sizeable number of RTKs (~21%) were predicted for caspase cleavage sites at the juxtamembrane region on the cytoplasmic side of the receptors (defined as the receptor segment between the transmembrane and kinase domains). Interestingly, it was reported that caspase cleavage of MET receptor at Asp^1000 ^results in the inactivation of functional MET receptor by loss of its signalling cytoplasmic domain, with the concomitant appearance of membrane bound MET and soluble intracellular MET fragments. The membrane-bound MET fragment prevents downstream survival activity by trapping its cognate ligand, while the intracellular MET fragment becomes ligand-independent. It is conceivable that caspase cleavage at the juxtamembrane sites on RTKs may lead to the truncation of the full length receptor into a membrane bound portion and an intracellular fragment, similar to the observation of MET cleavage. In addition, studies on caspase-cleaved RTKs suggest that intracellular RTK fragments may have downstream functional implications. The release of MET fragment containing the active kinase domain following caspase cleavage was shown to be pro-apoptotic in cells. Pro-apoptotic intracellular fragments were similarly observed downstream of caspase-mediated RET and Erbb2 cleavage as well. Observations on high-throughput proteomic screening of caspase substrates in Dix *et al*. [41] reported that a substantial number of caspase substrates are cleaved into persistently stable, domain-containing fragments, and further speculated that caspase-mediated proteolysis yields a class of effector protein fragments with novel functions. Also, caspase cleavage of ALK was found to unravel a pro-apoptotic intracellular region upstream of the cleavage site. Taken together, the presence of predicted juxtamembrane cleavage sites on the intracellular domain of the receptors indicate possible receptor cleavage which could lead to the interference of receptor signalling and the generation of pro-apoptotic signals.

On a related note, close to 80% (41/52) of all RTKs harbour caspase cleavage sites within the tyrosine kinase domain of the receptor. In particular, RTKs from the insulin receptor and FGF receptor sub-families are annotated with multiple cleavage sites within their tyrosine kinase domains. As these domains serve as key mediators of signal transduction for RTKs, structural alterations from caspase cleavage may lead to perturbations of downstream RTK signalling. Interestingly, studies by Tikhomirov *et al*. [42] indicate that proteolytic fragments bearing the motif "RLLGI" derived from the tyrosine kinase domains of EGFR, Erbb2, Erbb4, TrkA and VEGFR1 were able to induce apoptosis in cells. Indeed, caspase cleavage sites were predicted in the kinase domains of some of these receptors (EGFR; Asp^770^, Asp^916^, Erbb4; Asp^878^, Asp^922 ^and VEGFR1; Asp^958^, Asp^987 ^Asp^1135^), suggesting the possibility of caspase cleavage and release of pro-apoptotic intracellular kinase fragments. As the "RLLGI" motif is suggested to be prevalent among RTKs, it is possible that cleavage of the tyrosine kinase domains of several other RTKs could lead to the similar production of such pro-apoptotic fragments. Studies on Erbb2 cleavage have shown that caspase cleavage produced pro-apoptotic intracellular fragments downstream of the kinase domain in the C-terminal region of the receptor. Cleavage of EGFR at a comparable location was shown but no pro-apoptotic consequences were reported. Interestingly, the other members of the EGFR family, Erbb3 and Erbb4, were predicted to possess similarly located caspase cleavage sites, suggesting that these proteins could be caspase targets as well.

The presence and distribution of these predicted cleavage sites across the RTK family suggest a general role of caspase cleavage in regulating RTK function. It is tempting to speculate a phenomenon whereby caspase cleavage of RTKs leads to a molecular "life-death" switch which converts the pro-survival protein to a pro-apoptotic one through the exposure and/or the release of pro-apoptotic domains. The elegant integration of both anti- and pro-apoptotic functionalities on the same signalling protein is an uncommon but economical feature. As discussed in Fischer *et al*. [7], such dramatic reversal of protein function was similarly observed in the caspase cleavage of serine/threonine protein kinases, MEKK1 and MEKK4, which generated pro-apoptotic fragments upon cleavage at their kinase domains. Several other anti-apoptotic proteins such as Bcl-2 and Bcl-xl have been shown to be converted into pro-apoptotic molecules by caspase cleavage.

Intriguingly, most RTKs were predicted to harbour caspase cleavage sites on their extracellular domain. It is tempting to question if there are hitherto uncharacterized functional consequences of cleavage at these locations since all known substrate cleavage were reported to be localized only in the intracellular environment. Notably, active caspases were found to be released into the extracellular environment during apoptosis [43]. In addition, work by Cowan and co-workers [44] provided evidence for the localization of active caspase-2, caspase-3 and caspase-7 to the membrane surfaces of apoptotic smooth muscle cells. Clearly, future investigations on caspase activity in the extracellular environment will shed light on the possibility of extracellular RTK cleavage and its downstream consequences. More importantly, the predicted cleavage sites on RTKs will generate useful hypotheses and experimental leads for the validation and characterization of caspase-mediated RTK regulation.

## Discussion

In this paper, we propose a multi-factor model for the prediction of caspase substrates using a two-step approach. The entire protein sequence is first scanned for potential cleavage sites using a caspase cleavage sites prediction algorithm. The predicted cleavage sites will be filtered using a scoring system (given as the P-score) which is based on the propensities of predicted cleavage sites to locate in unstructured (C_p_) and solvent exposed regions (S_p_) on the protein. Expert domain knowledge or user requirements will direct the appropriate selection of the P-score cut-off levels. We have adopted the use of secondary structure and solvent accessibilities prediction tools as there are very limited experimentally verified structures on caspase substrates. As the model is dependent on the accuracy of existing secondary structure and solvent accessibility prediction tools, advancements in these domains will be helpful for this purpose.

Recently, the incorporation of additional factors, such as secondary structures and solvent accessibilities, was found to increase accuracy in HIV protease substrates prediction [45]. In that case, a three-level hierarchical classifier scans a protein sequence for HIV protease cleavage sites using specificity data and filters the output for sequences located within disorganized secondary structures and solvent exposed regions. These structural factors were similarly integrated with the neural network algorithms for RNA and DNA binding sites prediction and were found to be helpful [46,47]. In our proposed method, instead of combining the prediction of cleavage sites specificity, secondary structures and solvent accessibilities into a single predictor, these factors were accounted in two distinct steps to address a couple of caveats implicit in protease substrate prediction. While cleavage sites were shown to preferentially locate in unstructured and solvent exposed regions, not all predicted cleavage sites with substantial propensity for these factors will be cleaved *in vivo*. It is conceivable that regulatory processes such as post-translational modifications and other protein-protein interactions will likely to influence the final proteolytic event. Conversely, predicted cleavage sites which are hidden in deep hydrophobic cores of proteins - hence characterized by low propensities for solvent exposure - cannot be ruled out as it is possible that these sequences may be exposed following an upstream proteolytic cleavage of the protein by the same or another protease. Evidently, the caspase-mediated cleavage of ETK (epithelial and endothelial tyrosine kinase) was suggested to proceed in a two-step fashion where the first caspase cleavage site of the protein exposes an internal cleavage site for a subsequent round of cleavage [48]. The retinoblastoma protein, RB, the hepatocyte growth factor receptor, MET and GTP exchange factor for small G-protein Ras, RasGAP were all shown to be cleaved sequentially at multiple sites - further suggesting the possibility of structural changes following an upstream proteolytic cleavage event (reviewed in [7]). To circumvent these constraints, the proposed two-step model predicts for a broad pool of potential cleavage sites in the first step and filters the results through the P-score cut-off which can be appropriately assigned with expert domain knowledge or under different user requirements.

The two-step model was tested using two caspase cleavage site prediction methods - CASVM and GraBCas. It was shown that in both cases, the discrimination of predicted cleavage sites based on additional structural characteristics was helpful for reducing the false positives. The GraBCas-based model was shown to outperform the CASVM-based model by eliminating a greater percentage of false positives with full retention of true positives. A likely reason for the disparity in the results could be due to the different sequence windows used for prediction in each case. GraBCas requires only the tetrapeptide cleavage sequence, while a 24-mer peptide sequence (tetrapeptide sequence with flanking ten residues upstream and downstream) is needed for input into CASVM. Presumably, in the latter case, information encoded within factors for caspase cleavage site recognition would have overlapped to a greater extent or are more correlated with that for secondary structures and solvent exposure due to the longer sequence window. In any case, the results suggest that other cleavage sites prediction tools utilizing algorithms with low correlation with secondary structure and solvent accessibility prediction could be integrated into the model. Conversely, the addition of other factors with low correlations with cleavage site recognition would be helpful for improving prediction of substrate cleavage. Recent studies have suggested that exosites - or interaction sites distal from the enzyme active site - could mediate substrate cleavage and are responsible for non-canonical caspase substrate cleavage. Structural studies by Agniswarmy *et al*. [49] highlighted a symmetrical pentapeptide binding pocket on caspase-7 situated way from the active site which could function as an exosite. Exosites were also shown to be involved in proteolytic events mediated by blood coagulation proteases [50]. Similarly, it was reported that post-translational events such as serine phosphorylation of caspase cleavage sites, particularly on the P_4 _and P_1_' residues [51,52], and sumolyation [53] were inhibitory to substrate cleavage. It is likely that models incorporating these factors with existing caspase cleavage site prediction tools will enhance *in vivo *substrate prediction. As the prediction of other protease substrates is likely to be largely influenced by the set of factors similar to the ones suggested here, our proposed multi-factor model may be applicable to the prediction of other protease substrates given the required data.

## Conclusion

In this paper, we analyzed the structural characteristics of reported caspase substrates and found that caspase cleavage sites are more likely to locate in unstructured and solvent exposed regions compared to non-cleavage sites. We hypothesized that the integration of these factors with cleavage sites prediction will improve substrate prediction by filtering out predicted cleavage site sequences with unfavourable structural characteristics. Consequently, we constructed a two-step model integrating these factors with existing cleavage sites prediction tools. Using an independent dataset of caspase substrates, the model incorporating CASVM or GraBCas was shown to achieve greater positive predictive values compared to these methods alone, and was able to reduce the false positives pool by up to 13% and 53% respectively while retaining all true positives. As the prediction of other protease substrates is likely to be largely influenced by the set of factors similar to the ones suggested here, the multi-factor prediction model may be applicable to the prediction of other protease substrates.

Future progress in computational prediction of caspase substrates, and possibly for other protease-substrate system, will clearly hinge on the careful selection and integration of factors for substrate cleavage. It is certain that such efforts will be greatly assisted as more data, such as resolved structures of caspase substrates, becomes available. As high through-put screening efforts by Mahrus *et al*. [54] and Dix *et al*. [50] have uncovered several hundred more caspase substrates over the past year - an apparent indication of the burgeoning potential for future discovery of novel substrates - it is expected that *in silico *work will continue to complement experimental studies in the challenging journey of defining the caspase degradome on the systems-wide basis.

## Materials and methods

### Dataset

74 unique, experimentally verified cleavage sites were obtained from the dataset of caspase cleavage sites derived from Fischer *et al*. [7] (available in Additional File 3). 24-residue long subsequences comprising of the tetrapeptide sequence with flanking upstream 10 residues and downstream 10 residues (P_14_....P_4_P_3_P_2_P_1_......P_10_') were extracted from the respective substrates and assigned as "cleavage site" subsequences. One other tetrapeptide (not a verified cleavage site) was randomly selected on the respective substrate of each verified cleavage site and subsequences similar in length to the "cleavage site" subsequences were constructed. A total of 74 additional subsequences were constructed and designated as the "non-cleavage site" subsequences. Together, the pool of subsequences (148 in total) constitutes the analysis dataset and was used for the analysis of structural features and for the optimization of the prediction model parameters.

### Quantitative measures of secondary structures and solvent accessibilities

Each subsequence in the analysis dataset was predicted for secondary structures and solvent accessibilities using the SABLE II protein structure prediction server (using default parameters; server located at http://sable.cchmc.org) [55-57]. The SABLE server output was parsed with Perl scripts and quantitative measures of the propensities for helices (H_p_), *β*-strands (E_p_), coils (C_p_) and solvent accessibilities (S_p_) were computed using the following equations:

H_n_, E_n_, C_n _and S_n _are the SABLE predicted output for helix, *β*-strand, coil and real-value score (ranging 0 to 6; 0 for fully buried and 6 for maximum exposure) for solvent accessibility for each residue at position *n *in the sequence of length *N *(1, 2, 3.... *N*). S_max _is a constant with value of 144, which is the sum of real-value scores from SABLE II for all residues in the 24-mer subsequence assuming that each residue is maximally exposed to solvent (24 × 6 = 144).

P-score, the quantitative measure of the net propensities for both unstructured regions and solvent exposure on a subsequence is given as:

The P-score is the weighted sum of C_p _and S_p _values where the weights are given by the coefficients *α *and *β *respectively. Using the analysis dataset, values of *α *and *β *were optimized to 0.3 and 0.7 respectively. Optimal *α *and *β *coefficient values were obtained by stepping through various combinations of values (0.0, 0.1, 0.2....1.0), and measuring the fraction of cleavage site subsequences and non-cleavage site subsequences retained at increasing P-score cut-offs (details are available in the Additional File 4).

### Multi-factor model testing

For model testing, a test dataset of unique caspase cleavage sites (14 caspase substrates containing 17 unique cleavage site sequences; available in Additional File 3) was used. The test dataset was predicted for caspase cleavage sites using CASVM (P_14_P_10_' scanning window and P_1 _residue (Asp) options were selected) or GraBCas (cleavage sites were scored using the GraBCas matrices and the highest score was selected; cut-off of 0.1 was used). Predicted caspase cleavage sites were extracted from substrate sequences and 24-mer subsequences comprising of the predicted tetrapeptide sequences with flanking upstream ten residues and downstream ten residues (P_14_....P_4_P_3_P_2_P_1_......P_10_') were constructed and calculated for S_p_, C_p _and P-score values. Subsequences containing the cleavage sites were assigned as "true positives" while those containing non-cleavage sites were denoted as "false positives". Percentage of subsequences from both pools retained at each P-score cut-off (0.00, 0.05, 0.10...1.00) and corresponding positive predictive values (PPV) were calculated for both models. In this context, PPV measures the probability of hitting a true cleavage site when restricted to all predicted cleavage sites and is computed as TP/(TP+FP), where TP and FP are the number of "true positives" and "false positives" respectively.

## Competing interests

The authors declare that they have no competing interests.

## Authors' contributions

LJKW conceived the two-step, multi-factor model for prediction of caspase substrates. TWT and SR contributed with ideas on the experimentation and assisted with the drafting of the manuscript. All authors read and approved the final manuscript.

## Note

Other papers from the meeting have been published as part of *BMC Bioinformatics *Volume 10 Supplement 15, 2009: Eighth International Conference on Bioinformatics (InCoB2009): Bioinformatics, available online at http://www.biomedcentral.com/1471-2105/10?issue=S15.

## Supplementary Material

Additional file 1 **Matrix showing global mapping of reported caspase cleavage sites across all known substrates**. Each cell in table shows the frequency of occurrence of an experimentally verified caspase cleavage site sequence (column) in a known substrate (row). Coloured cell indicates frequency of occurrence of that sequence in the substrate (yellow, 1; orange, 2; red, 3 and above). Click here for file

Additional file 2 **Global mapping of predicted caspase cleavage sites on receptor tyrosine kinases**. Positions of CASVM predicted cleavage sites on protein sequence of each RTK member are listed. Numbers indicate the positions of P1 (Asp) residues on protein sequences. All cleavage site positions are color coded; grey indicates location of cleavage site within the extracellular domain, blue indicates location within intracellular domain and darker blue indicates location within kinase domain (all kinase domains of RTKs are located in the intracellular domain of the receptor). Predicted cleavage sites corresponding to true experimentally verified cleavage sites on EGFR, ERBB2, MET, ALK and RET are underlined. Yellow highlights indicate predicted cleavage sites with P-score of 0.3 or smaller. Click here for file

Additional file 3 **Analysis dataset of caspase substrate cleavage sites**. List of caspase substrate cleavage sites used for the analysis of structural features and for the optimization of the prediction model parameters. Cleavage sites are reported as tetrapeptides in the order: P4-P3-P2-P1. All cleavage sites have an Asp (D) in the P1 position. The position of the P1 amino acid in the protein sequence is given as reported in Uniprot. Click here for file

Additional file 4 **Optimization of *α *and *β *coefficients in the P-score function**. Pools of cleavage site subsequences (74) and non-cleavage site subsequences (74) were assigned with P-score values using different combinations of *α *and *β *coefficient values (1.0 to 0.0 and 0.0 to 1.0 respectively). The two pools were measured for the fraction of subsequences (vertical axis) with scores above the P-score cut-offs (horizontal axis) (blue line: cleavage site subsequences, red line: non-cleavage site subsequences, green line: all subsequences) using the different combinations of *α *and *β *coefficients (Figures A-K). The values of 0.3 and 0.7 were selected for *α *and *β *coefficients respectively as the resultant P-score function produced the best combination of cleavage site subsequences retention and elimination of non-cleavage site subsequences under increasing P-score cut-offs. Click here for file
