# Supplementary material for: A multi-factor model for caspase degradome prediction
Source: BMC Genomics. 2009 Dec 3;10(Suppl 3):S6. doi: 10.1186/1471-2164-10-S3-S6 (PMC2788393; doi:10.1186/1471-2164-10-S3-S6)
Supplement: Additional file 2 — Global mapping of predicted caspase cleavage sites on receptor tyrosine kinases. Positions of CASVM predicted cleavage sites on protein sequence of each RTK member are listed. Numbers indicate the positions of P1 (Asp) residues on protein sequences. All cleavage site positions are color coded; grey indicates location of cleavage site within the extracellular domain, blue indicates location within intracellular domain and darker blue indicates location within kinase domain (all kinase domains of RTKs are located in the intracellular domain of the receptor). Predicted cleavage sites corresponding to true experimentally verified cleavage sites on EGFR, ERBB2, MET, ALK and RET are underlined. Yellow highlights indicate predicted cleavage sites with P-score of 0.3 or smaller. [file 1471-2164-10-S3-S6-S2.pdf]

A multi-factor model for caspase degradome prediction by Lawrence J.K. Wee, Tin Wee Tan, Shoba Ranganathan

Additional File 2: Global mapping of predicted caspase cleavage sites on receptor tyrosine kinases

RTK Family	RTKs	UNIPROT ID	Predicted Caspase Cleavage Sites[1]														
EGF receptor	EGFR	P00533	321	458	587	770	916	1006	1009	1012	1083	1127	1152	1171			
	ERBB2	P04626	277	326	382	639	1016	1019	1087	1125							
	ERBB3	P21860	162	165	242	581	1010	1020	1327								
	ERBB4	Q15303	218	245	300	335	510	564	585	595	878	922	1012	1015	1018	1068	1241
Insulin receptor	INSR	P06213	75	483	526	546	549	672	704	716	949	985	1145	1210	1259	1330	1344
	INSRR	P14616	154	585	676	816	916	1101	1166	1207	1280						
	IGF1R	P08069	156	300	342	519	539	542	675	1121	1186	1235	1294	1306			
	ROS1	P08922	100	358	483	513	684	711	842	1202	1391	1853	2058	2062	2135	2247	
PDGF receptor	PDGFRA	P16234	215	244	287	422	568	733	763	846	902	919	1015	1024	1033	1074	
	PDGFRB	P09619	78	200	285	575	691	737	1091								
	CSF1R	P07333	51	63	269	741	746										
	KIT	P10721	439	479	768												
	FLT3	P36888	200	455	600	959											
FGF receptor	FGFR1	P11362	69	90	110	130	131	132	133	142	218	527	768	782			
	FGFR2	P21802	75	126	135	136	138	506	521	530	785	794	795				
	FGFR3	P22607	77	136	139	143	147	497	516	521	776	792					
	FGFR4	P22455	119	129	187	240	507	516	575	770	779						

RTK Family	RTKs	UNIPROT ID	Predicted Caspase Cleavage Sites[1]												
VEGF receptor	VEGFR1	P17948	372	495	630	958	987	1135	1165	1168	1262				
	VEGFR3	P35916	19	45	77	304	371	556	725	728	1130	1216	1274		
	VEGFR2	P35968	173	180	295	392	639	717	852	1141	1171	1174	1189	1259	1315
HGF receptor	MET	P08581	174	231	352	824	1002	1231	1376	1380					
	MST1R	Q04912	126	176	204	299	355	375	671	805	927	936	1030	1045	1235
TRK receptor	TRKA	P04629	53	209	306										
	TRKB	Q16620	173	277	349	406	409	424	476	579					
	TRKC	Q16288	61	193	476	641									
EPH receptor	EPHA1	P21709	32	45	158	252	592	778	841						
	EPHA2	P29317	33	232	250	708									
	EPHA3	P29320	17	34	159	282	299	531	708						
	EPHA4	P54764	35	161	241	319	402	542							
	EPHA5	P54756	65	187	190	348	430	995							
	EPHA6	Q9UF33	57	163	243	440	970								
	EPHA7	Q15375	37	314	404										
	EPHA8	P29322	55	61	222	241	729	790	940						
	EPHB1	P54762	24	118	138	528	771	840							
	EPHB2	P29323	25	139	318	774	777								
	EPHB3	P54753	138	333	785	786	918	935							
	EPHB4	P54760	226	242	836										
	EPHB6	O15197	63	139	142	271									

RTK Family	RTKs	UNIPROT ID	Predicted Caspase Cleavage Sites[1]									
AXL receptor	AXL	P30530	87	260	270	389	407	551	648	769	843	
	TYRO3	Q06418	73	79	121	250	335	576	638	763		
	MERTK	Q12866	309	402	610	706	827	843	900	983		
LTK receptor	LTK	P29376	87	193	335	340	348	557	705			
	ALK	Q9UM73	305	516	885	951	954	993	1017	1163	1311	1606
TIE receptor	TIE1	P35590	287	391	474	560	578	586	861	883		
	TIE2	Q02763	137	357	389	473	663	846	868	923		
ROR receptor	ROR1	Q01973	167	387	395	580	591					
	ROR2	Q01974	42	51	262	390	903					
DDR receptor	DDR1	Q08345	44	46	68	70	189	216	598	604	630	729
	DDR2	Q16832	69	125	189	234	240					
RET receptor	RET	P07949	43	264	267	547	567	707	797	1017	1031	
RYK receptor	RYK	P34925	258	351	554							
MuSK receptor	MUSK	O15146	94	474	622	743	817					

[1] Positions of CASVM predicted cleavage sites on protein sequence of each RTK member are listed. Numbers indicate the positions of P_1 (Asp) residues on protein sequences. All cleavage site positions are color coded; grey indicates location of cleavage site within the extracellular domain, blue indicates location within intracellular domain and darker blue indicates location within kinase domain (all kinase domains of RTKs are located in the intracellular domain of the receptor). Predicted cleavage sites corresponding to true experimentally verified cleavage sites on EGFR, ERBB2, MET, ALK and RET are underlined. Yellow highlights indicate predicted cleavage sites with P-score of 0.3 or smaller.
