# Supplementary material for: A multi-factor model for caspase degradome prediction
Source: BMC Genomics. 2009 Dec 3;10(Suppl 3):S6. doi: 10.1186/1471-2164-10-S3-S6 (PMC2788393; doi:10.1186/1471-2164-10-S3-S6)
Supplement: Additional file 3 — Analysis dataset of caspase substrate cleavage sites. List of caspase substrate cleavage sites used for the analysis of structural features and for the optimization of the prediction model parameters. Cleavage sites are reported as tetrapeptides in the order: P4-P3-P2-P1. All cleavage sites have an Asp (D) in the P1 position. The position of the P1 amino acid in the protein sequence is given as reported in Uniprot. [file 1471-2164-10-S3-S6-S3.pdf]

A multi-factor model for caspase degradome prediction

by Lawrence J.K. Wee, Tin Wee Tan, Shoba Ranganathan

Additional File 3: Analysis dataset of caspase substrate cleavage sites

Caspase Substrate	Uniprot Accession ID	Cleavage Site[1]	P_1 Position[2]
Apaf-1	O14727	SVTD	271
Bad	Q92934	EQED	14
Bax	Q07812	FIQD	33
Bcl-2	P10415	DAGD	34
Bcl-xL	Q07817	HLAD	61
Bid	P55957	LQTD	60
c-FLIP	O15519	LEVD	376
c-IAP1	Q13490	ENAD	372
XIAP	P98170	SESD	242
APC	P25054	DNID	777
Cas	Q63767	DVPD	416
		DSPD	748
β-Catenin	P35222	SYLD	32
		ADID	83
		TQFD	115
		YPVD	751
Desmoglein-3	P32926	DYAD	781
E-cadherin	P12830	DTRD	750
P-cadherin	P22223	DTRD	696
FAK	Q05397	DQTD	772
HEF1	Q14511	DLVD	363
		DDYD	630
Connexin 45.6	P36383	DEVE	367
Paxillin	Q8VI37	NTQD	102
β-Actin	P60709	ELPD	244
α-Adducin	P35611	DDSD	633
CD-IC	O88485	DSGD	99
α-II-Fodrin	Q13813	DETD	1185
β-II-Fodrin	Q01082	DEVD	1457
Gas2	O43903	SRVD	278
Gelsolin	P06396	DQTD	403
HIP-55	Q6IAI8	EHID	361
Cytokeratin 18	P05783	VEVD	237
		DALD	396
vMLC	P08590	DFVE	134
β-II Spectrin	Q01082	DEVD	1457
		ETVD	2146
Troponin T	P45379	VDFD	97

Caspase Substrate	Uniprot Accession ID	Cleavage Site[1]	P_1 Position[2]
Vimentin	P08670	DSVD	84
Lamin A	P02545	VEID	230
Lamin B1	P20700	VEVD	230
Lamin C	P02545-2	VEID	230
LAP2-α	P42166	KRID	412
Nup153	P49790	DITD	349
SAF-A	Q00839	SALD	100
SATB1	Q01826	VEMD	254
Tpr	P12270	DSQD	1892
p28BAP31	P51572	AAVD	163
GRASP65	Q91X51	SLLD	319
c-Abl	P00519	DTTD	546
Cdc6	Q99741	LVFD	99
Cyclin A2	P18606	DEPD	90
MDM2/HDM2	Q00987	DVPD	361
MDMX	O15151	DVPD	361
NuMA	Q14980	DSLD	1726
p21Waf	P38936	DHVD	112
p27Kip1	P46527	DPSD	139
Rb	P06400	DEAD	886
Acinus	Q9UKV3	DELD	1093
ATM	Q13315	DYPD	863
BLM	P54132	TEVD	415
BRCA-1	P38398	DLLD	1155
ICAD	O00273	DETD	117
Helicad	Q8R5F7	DNTD	208
PARG	Q86W56	DEID	256
PARP-1	P09874	DEVD	213
PARP-2	O88554	LQMD	187
Rad21	O60216	DSPD	279
Rad51	Q06609	DVLD	187
RFC140	P35251	DEVD	723
Topo I	P11387	PEDD	123
AP-2 α	P05549	DRHD	19
CREB	P16220	ILND	140
GATA-1	P15976	EGLD	42

1. Cleavage sites are reported as tetrapeptides in the order: P_4-P_3-P_2-P_1. All cleavage sites have an Asp (D) in the P_1 position.
2. Indicates the position of the P_1 amino acid in the protein sequence as reported in Uniprot.

Test dataset of caspase substrate cleavage sites

Caspase Substrate	Uniprot Accession ID	Cleavage Site[1]	P_1 Position[2]
AP-1 complex (γ-adaptin)	P22892	DMTD	746
BAT3	P46379	DEQD	1001
CEACAM1-L	P31809	DQRD	460
CTEN	Q8IZW8	DSTD	570
DIAP1	Q24306	DQVD	20
Her-2	P04626	DVFD	1087
JNK 1 β2	P45983-4	SDTD	413
MITF	O75030-9	DLTD	345
NDUSF1 (p75 subunit of complex1)	P28331	DVMD	255
p23 co-chaperone	Q15185	DGAD	145
		PEVD	142
p65	Q04206	DCRD	97
PTEN	P60484	DVSD	371
		NEPD	375
		QEID	301
Rad9	Q6FI29	DDID	304
SCL/Tal-1	P17542	EITD	180

1. Cleavage sites are reported as tetrapeptides in the order: P_4-P_3-P_2-P_1. All cleavage sites have an Asp (D) in the P_1 position.
2. Indicates the position of the P_1 amino acid in the protein sequence as reported in Uniprot.
