# Supplementary material for: A multi-factor model for caspase degradome prediction
Source: BMC Genomics. 2009 Dec 3;10(Suppl 3):S6. doi: 10.1186/1471-2164-10-S3-S6 (PMC2788393; doi:10.1186/1471-2164-10-S3-S6)
Supplement: Additional file 4 — Optimization of α and β coefficients in the P-score function. Pools of cleavage site subsequences (74) and non-cleavage site subsequences (74) were assigned with P-score values using different combinations of α and β coefficient values (1.0 to 0.0 and 0.0 to 1.0 respectively). The two pools were measured for the fraction of subsequences (vertical axis) with scores above the P-score cut-offs (horizontal axis) (blue line: cleavage site subsequences, red line: non-cleavage site subsequences, green line: all subsequences) using the different combinations of α and β coefficients (Figures A-K). The values of 0.3 and 0.7 were selected for α and β coefficients respectively as the resultant P-score function produced the best combination of cleavage site subsequences retention and elimination of non-cleavage site subsequences under increasing P-score cut-offs. [file 1471-2164-10-S3-S6-S4.pdf]

A multi-factor model for caspase degradome prediction

by Lawrence J.K. Wee, Tin Wee Tan, Shoba Ranganathan

Additional File 4: β coefficients in the P-score function

Pools of cleavage sites (74) and non-cleavage sites (74) were assigned with P-score values using different combinations of α and β coefficient values (1.0 to 0.0 and 0.0 to 1.0 respectively). The two pools were measured for the fraction of sequences (*vertical axis*) with scores above the P-score cut-offs (*horizontal axis*) (*blue line*: cleavage site sequences, *red line*: non-cleavage site sequences, *green line*: all sequences) using the different combinations of α and β coefficients (Figures A-K). The values of 0.3 and 0.7 were selected for α and β coefficients respectively as the resultant P-score function produced the best combination of cleavage site sequences retention and elimination of non-cleavage site sequences under increasing P-score cut-offs.

A.

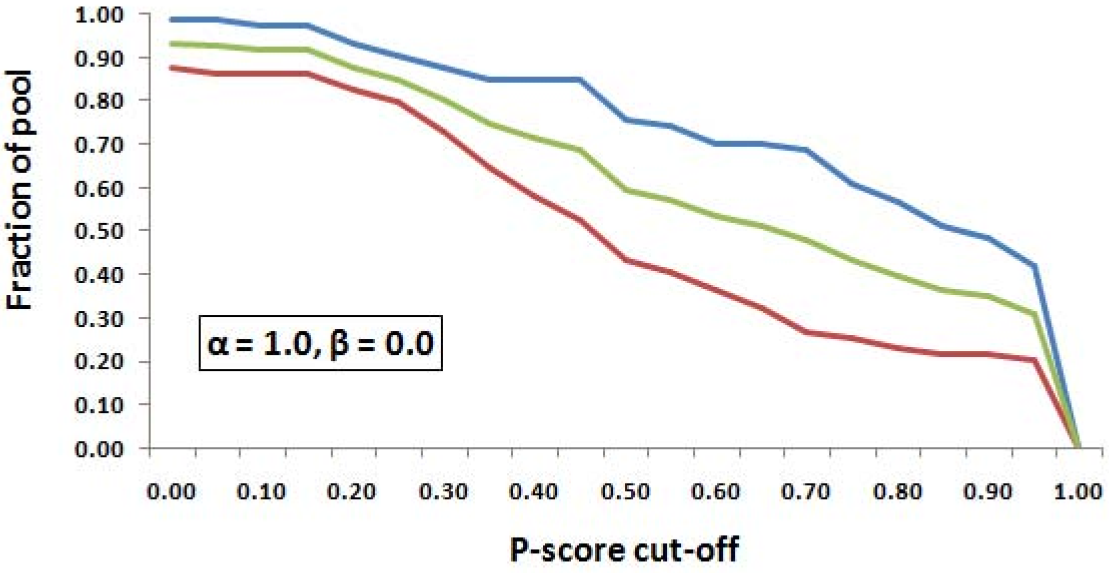

B.

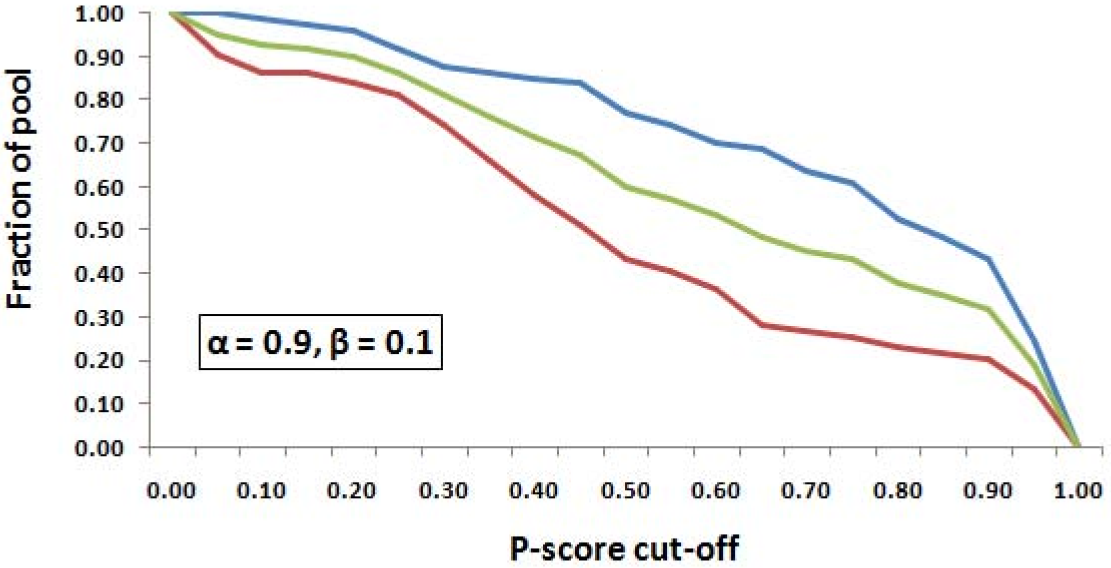
1.00
0.90
0.80
0.70
0.60
0.50
0.40
0.30
0.20
0.10
0.00
Fraction of pool
α = 0.9, β = 0.1
0.00
0.10
0.20
0.30
0.40
0.50
0.60
0.70
0.80
0.90
1.00
P-score cut-off

C.

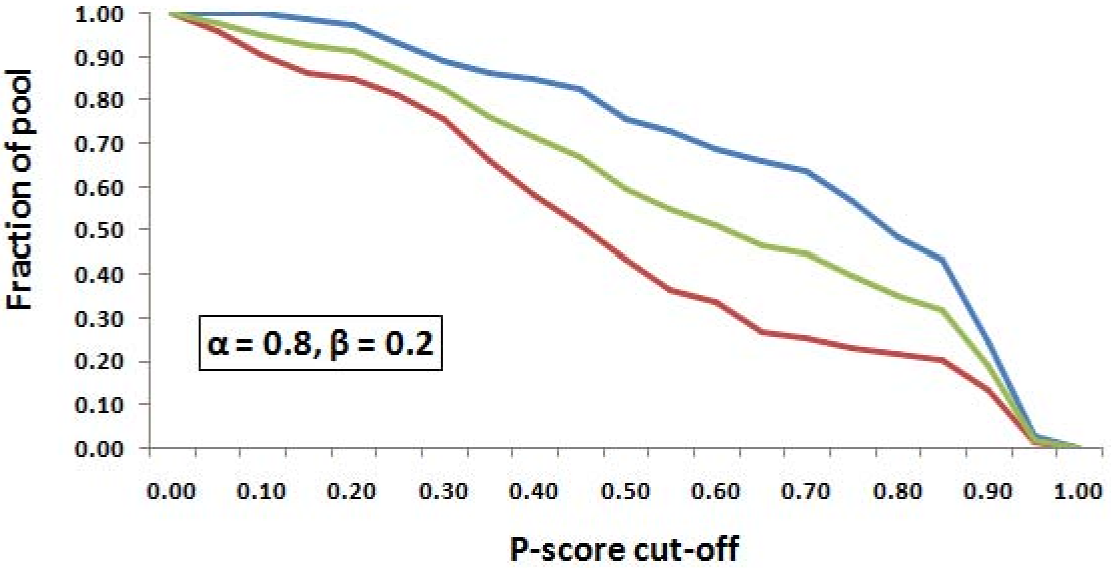
1.00
0.90
0.80
0.70
0.60
0.50
0.40
0.30
0.20
0.10
0.00
Fraction of pool
α = 0.8, β = 0.2
0.00
0.10
0.20
0.30
0.40
0.50
0.60
0.70
0.80
0.90
1.00
P-score cut-off

D.

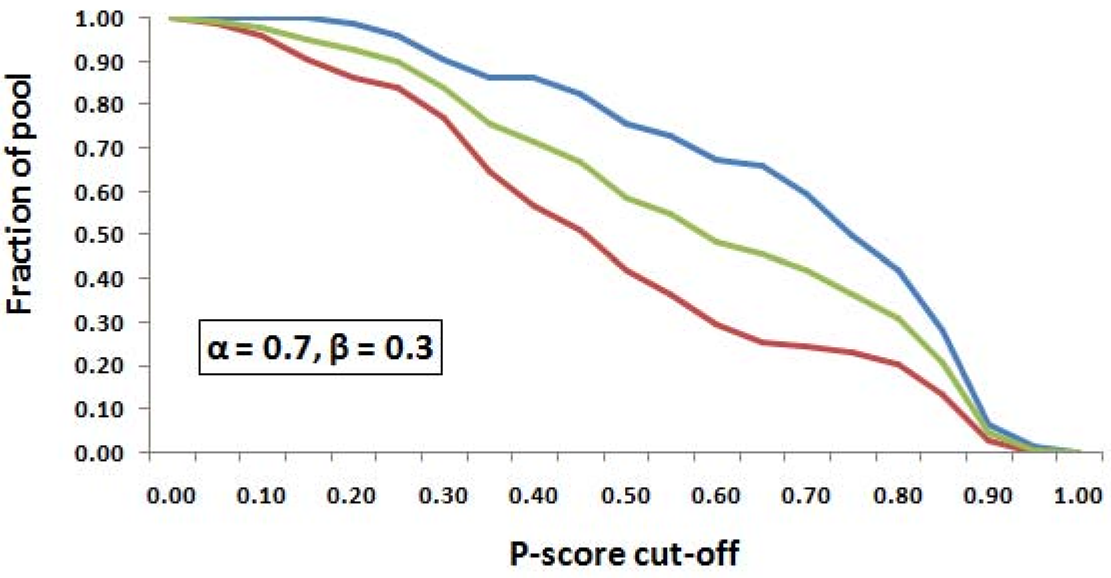
Fraction of pool
1.00
0.90
0.80
0.70
0.60
0.50
0.40
0.30
0.20
0.10
0.00
α = 0.7, β = 0.3
0.00
0.10
0.20
0.30
0.40
0.50
0.60
0.70
0.80
0.90
1.00
P-score cut-off

E.

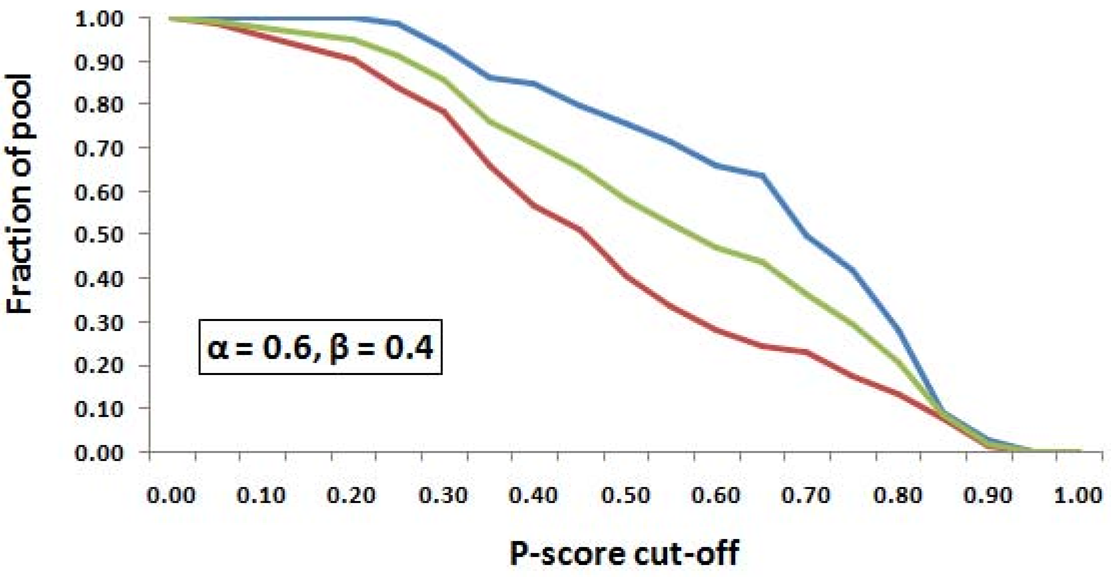
Fraction of pool
1.00
0.90
0.80
0.70
0.60
0.50
0.40
0.30
0.20
0.10
0.00
α = 0.6, β = 0.4
0.00
0.10
0.20
0.30
0.40
0.50
0.60
0.70
0.80
0.90
1.00
P-score cut-off

F.

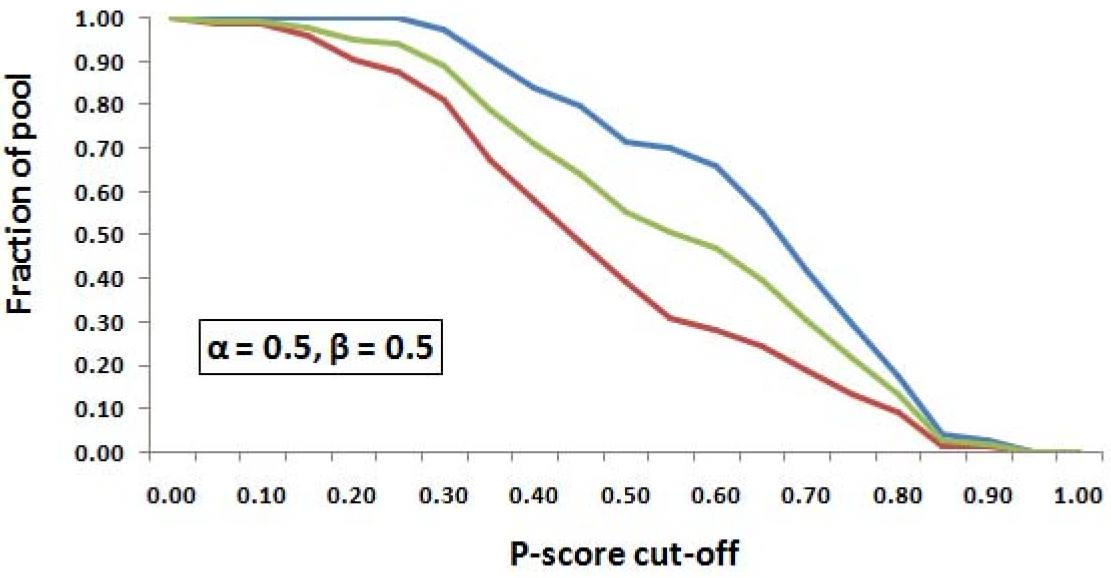
1.00
0.90
0.80
0.70
0.60
0.50
0.40
0.30
0.20
0.10
0.00
Fraction of pool
α = 0.5, β = 0.5
0.00
0.10
0.20
0.30
0.40
0.50
0.60
0.70
0.80
0.90
1.00
P-score cut-off

G.

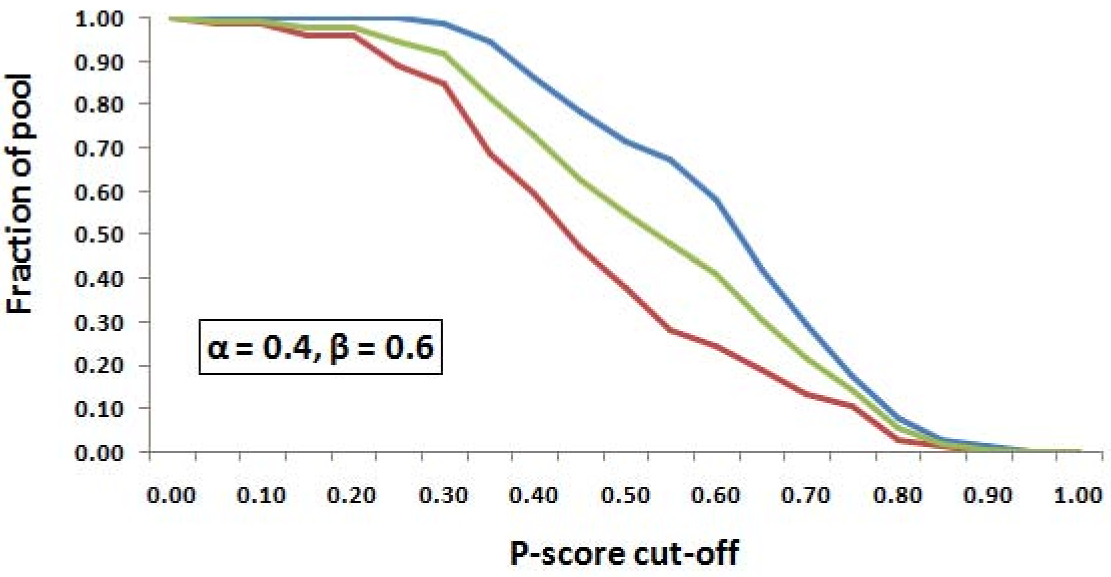
1.00
0.90
0.80
0.70
0.60
0.50
0.40
0.30
0.20
0.10
0.00
Fraction of pool
α = 0.4, β = 0.6
0.00
0.10
0.20
0.30
0.40
0.50
0.60
0.70
0.80
0.90
1.00
P-score cut-off

H.

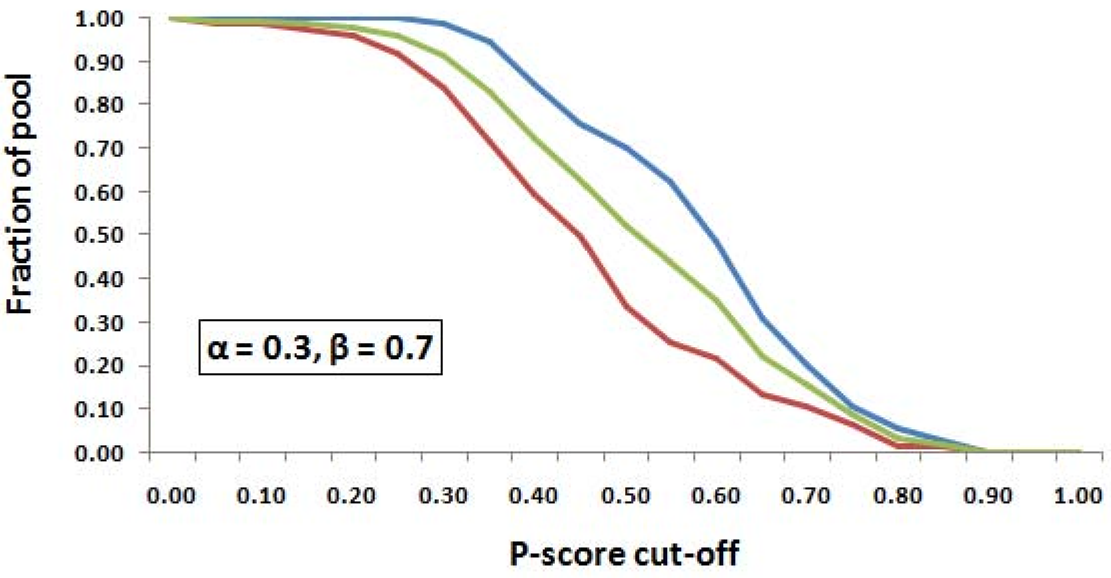
Fraction of pool
1.00
0.90
0.80
0.70
0.60
0.50
0.40
0.30
0.20
0.10
0.00
α = 0.3, β = 0.7
0.00
0.10
0.20
0.30
0.40
0.50
0.60
0.70
0.80
0.90
1.00
P-score cut-off

I.

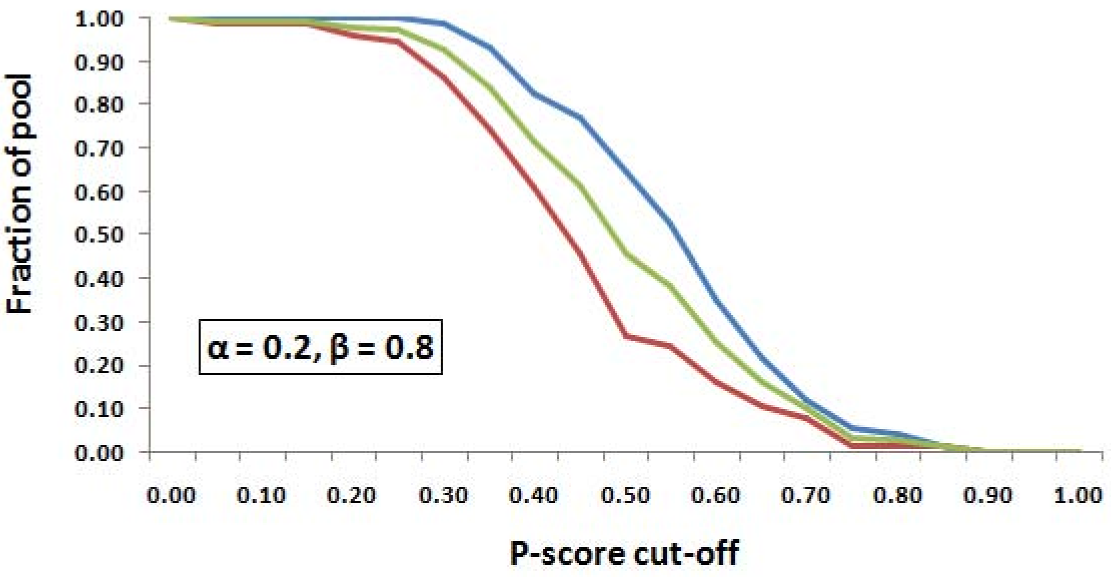
Fraction of pool
1.00
0.90
0.80
0.70
0.60
0.50
0.40
0.30
0.20
0.10
0.00
α = 0.2, β = 0.8
0.00
0.10
0.20
0.30
0.40
0.50
0.60
0.70
0.80
0.90
1.00
P-score cut-off

J.

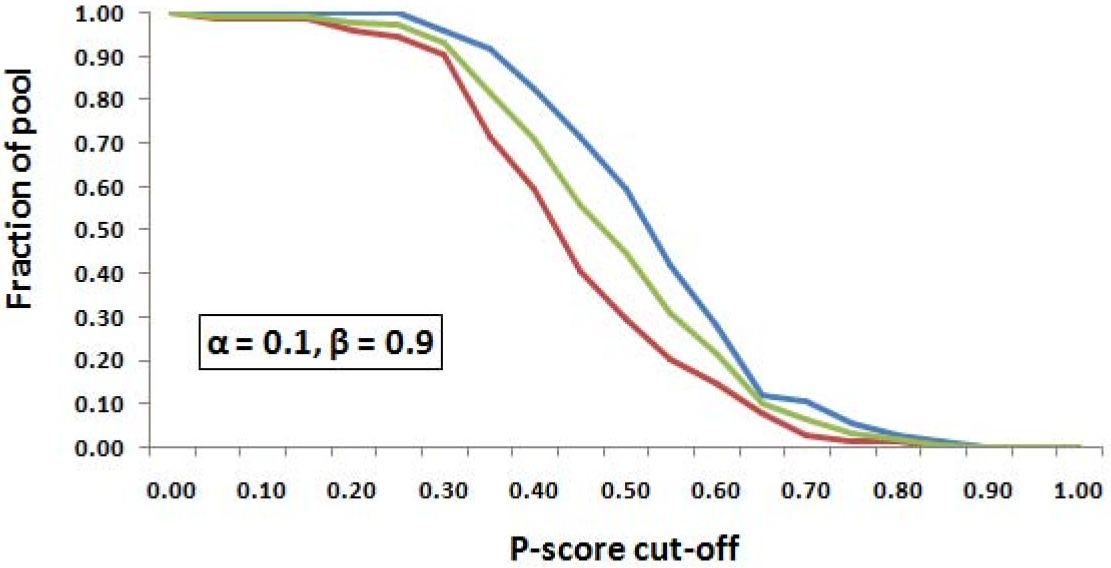
1.00
0.90
0.80
0.70
0.60
0.50
0.40
0.30
0.20
0.10
0.00
Fraction of pool
α = 0.1, β = 0.9
0.00
0.10
0.20
0.30
0.40
0.50
0.60
0.70
0.80
0.90
1.00
P-score cut-off

K.

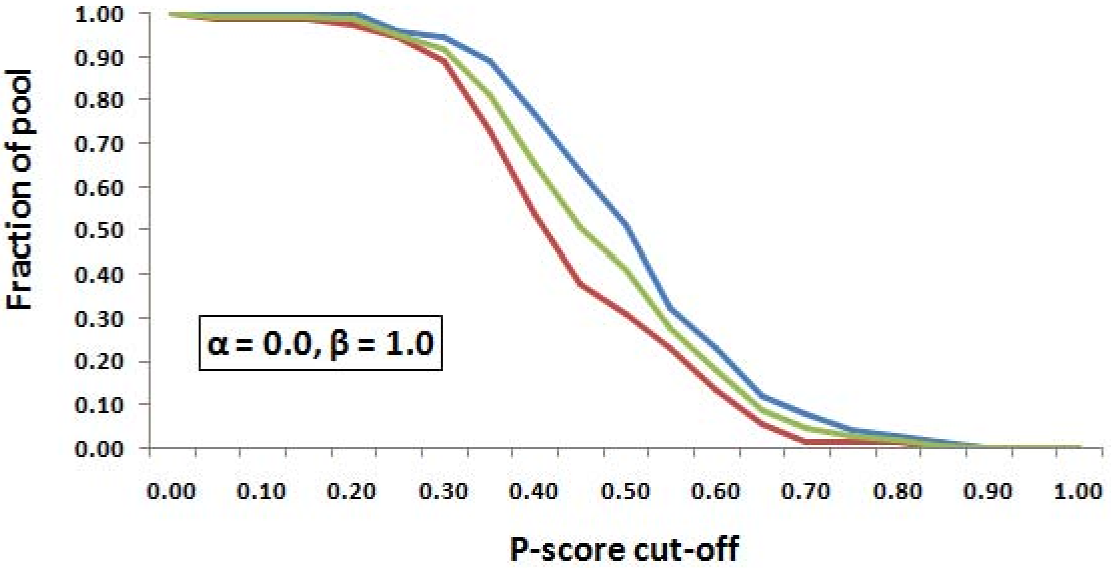
1.00
0.90
0.80
0.70
0.60
0.50
0.40
0.30
0.20
0.10
0.00
Fraction of pool
α = 0.0, β = 1.0
0.00
0.10
0.20
0.30
0.40
0.50
0.60
0.70
0.80
0.90
1.00
P-score cut-off
